# Supplementary material for: FACS-based dual fluorescence reporter assay demonstrates efficacy of antisense oligonucleotide therapy of novel PRPF3 intronic splice variant
Source: Mol Ther Nucleic Acids. 2026 May 8;37(2):102949. doi: 10.1016/j.omtn.2026.102949 (PMC13213770; doi:10.1016/j.omtn.2026.102949)
Supplement: Document S1. Figures S1–S6 and Tables S1–S4 [file mmc1.pdf]

OMTN, Volume *37*

Supplemental information

FACS-based dual fluorescence reporter assay demonstrates efficacy of antisense oligonucleotide therapy of novel *PRPF3* intronic splice variant

Vadim Dolgin, Ekaterina Eremenko, Ginat Narkis, Masha Mazor-Oring, Libe Gradstein, and Ohad S. Birk

Supplemental Figures and Tables

Table S1. Full-field ERG results in Individual III-1 at age 18.

		OD	OS	Reference
Light-adapted 3.0 ERG	b-wave: amplitude, μv<br>b-wave: latency, msec	14<br>32	17<br>32	Min 85<br>Max 32
Light-adapted 3.0 flicker	amplitude, μv<br>latency, msec	-<br>-	-<br>-	Min 65<br>Max 30
Dark-adapted blue light ERG	Rod response, μv	8	-	Min 60
Dark-adapted red light ERG	Cone response, μv	12	10	Min 50
Dark-adapted 3.0 ERG	a-wave, μv<br>a-wave, μv	4<br>6	3<br>6	Min 120<br>Min 280
Dark-adapted 30 ERG	a-wave, μv<br>a-wave, μv	2<br>7	4<br>6	Min 210<br>Min 320

Table S2. ACMG criteria for variant *PRPF3*: c.1527-23G>A (NM_004698.4) classification.

Criteria	PM2 sup	PM4 sup	PP1 strong	PP4 sup	PP3 strong
Notes	Allele frequency is extremely low	Protein length changes resulting from in-frame insertion in a non-repeat region	Cosegregation with disease in multiple affected family members in a gene definitively known to cause the disease	Patient's phenotype or family history is highly specific for a disease with a single genetic etiology.	Computational prediction tools unanimously support a deleterious effect on the gene

Table S3. AON sequences.

AON 1	AGAUCAGAGGCAGGGCUGAGG
AON 2	UGAAGAUCAGAGGCAGGGCUGA
AON 3	UGAAGAUCAGAGGCAGGGCU
AON 4	GCAUUGAAGAUCAGAGGCAG
AON 5	GGCAUUGAAGAUCAGAGGCA
AON 6	UGAGAAAGGCAUUGAAGAUCAGAGGCAGGGCU
AON 7	AUC**U**ACAAGUGAGAAAGGCAUUGAAGAUC
AON 8	GUGACAGGAAGGACAUC**U**AC
SCRAMBLED1	AUGGCAUUGAAGCUACAAGUGAGAAAAUC
SCRAMBLED2	AUCUACAAGUGAGAAAAGUUACGGAGAUC
OFTARGET1	GCAUCAUCAUUACUACUACG
OFTARGET2	GAUACGAUACAGACAUAUAC
MISMATCH1	AUCCACAAUUGAAAAAGUCAUUGAAGAUC
MISMATCH2	AUCUUCAAGUGAGACAGGCACUGAAUAUC

Table S4. Primers used.

Primer name	Sequence from 5’ to 3’
g*PRPF3*-F	GGTGGAAACCTCGGGCAAGTA
g*PRPF3*-R	AGTAGGAACTGGTGTTAGGTATGCT
cDNA-*PRPF3*-F	CTCCAGAACCCAAAGTGAGAATTTC
cDNA-*PRPF3*-R	GTACAGTTGCCCAGCATTGG
FMv2_vec-F	TGTATAAGTAATTTAAACCGCTGATCAGCCTCG
FMv2_vec-R	CTCACCATCAGCTTGGGTCTCCCTATAGTGAG
FMv2_ins-F	CCAAGCTGATGGTGAGCAAGGGCGAG
FMv2_ins-R	CAGCGGTTTAAATTACTTATACAGTTCGTCCATGCCGAG
MUT-FMv2_*PRPF3*-F	TCTCACTTGTaGATGTCCTTC
MUT-FMv2_*PRPF3*-R	AAGGCATTGAAGATCAGAG
NOT1-FMpMP-F	aaaaagcggcCGCCACCATGGTGAGCAAG
EcoRI-FMpMP-R	aaaaaagaatTCGAGTTACTTATACAGTTCGTCCATGCCG
u7ext-F	CTGGCTAGCTAACAACATAGGAGCTG
u7ext-R	GAAACCAGAGAAGGATCAAAGCCC
u7int-F	CTACAGACGCACTTCCGCAA
u7int-R	GAAGTCAGAAAACCTGCTCCAAAAATT
GAPDH-F	TCAACAGCGACACCCACTCC
GAPDH-R	AGCCAAATTCGTTGTCATACCAGG
GFP-F5	AGTTTGAGGGTGACACACTC
GFP-R3	CCATGATATACACGTTATGGGAGTTGTAGTT

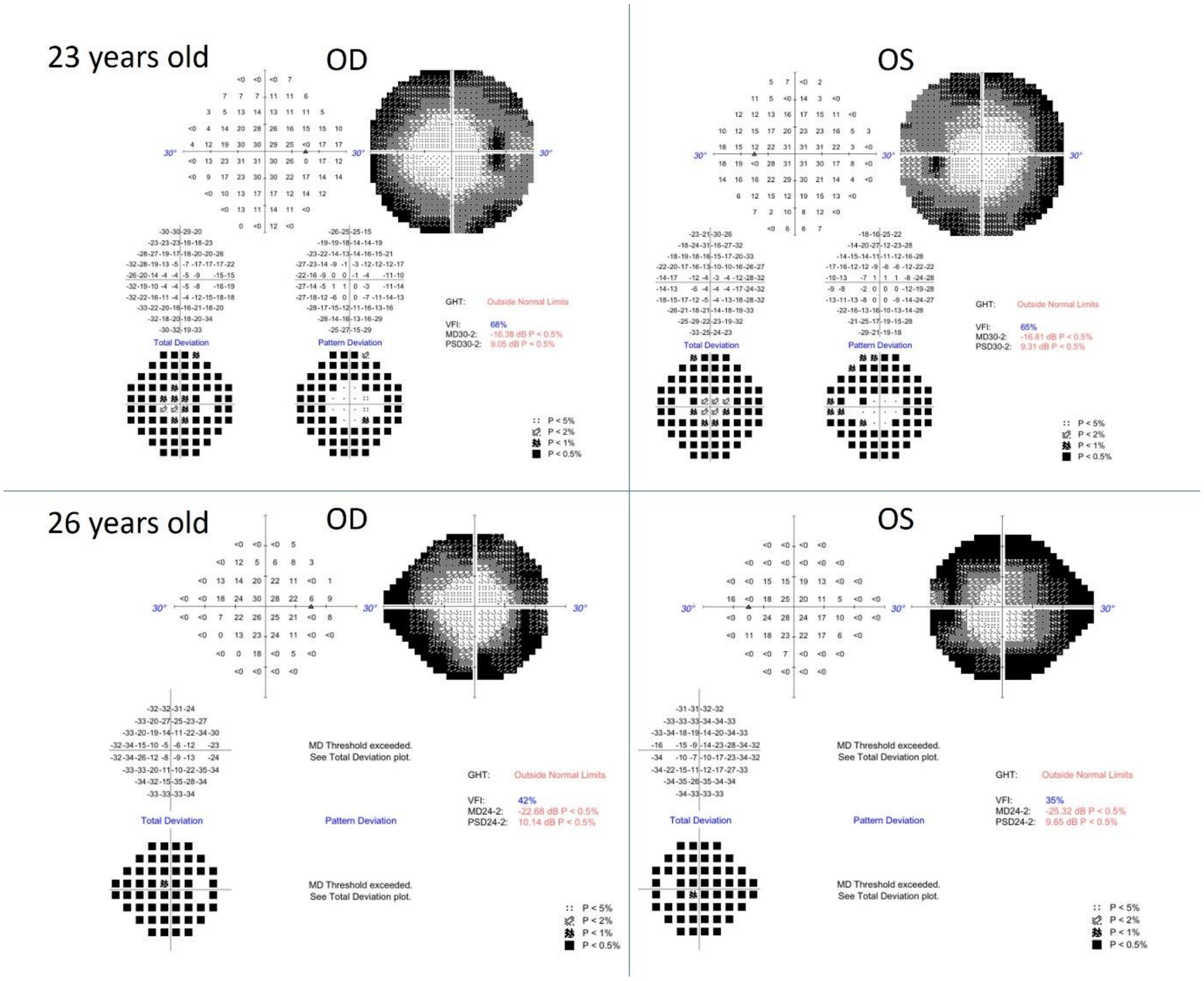

Fig. S1. Single Field Analysis of Visual Fields in Individual III-3 at ages 23 and 26. OD – right eye, OS – left eye. Classic visual field defects associated with RP: peripheral vision loss, evident as darkened areas on the perimetry map, indicating significant deficits in the retinal sensitivity typical for RP. Central vision is relatively preserved, highlighting the progressive nature of the disease.

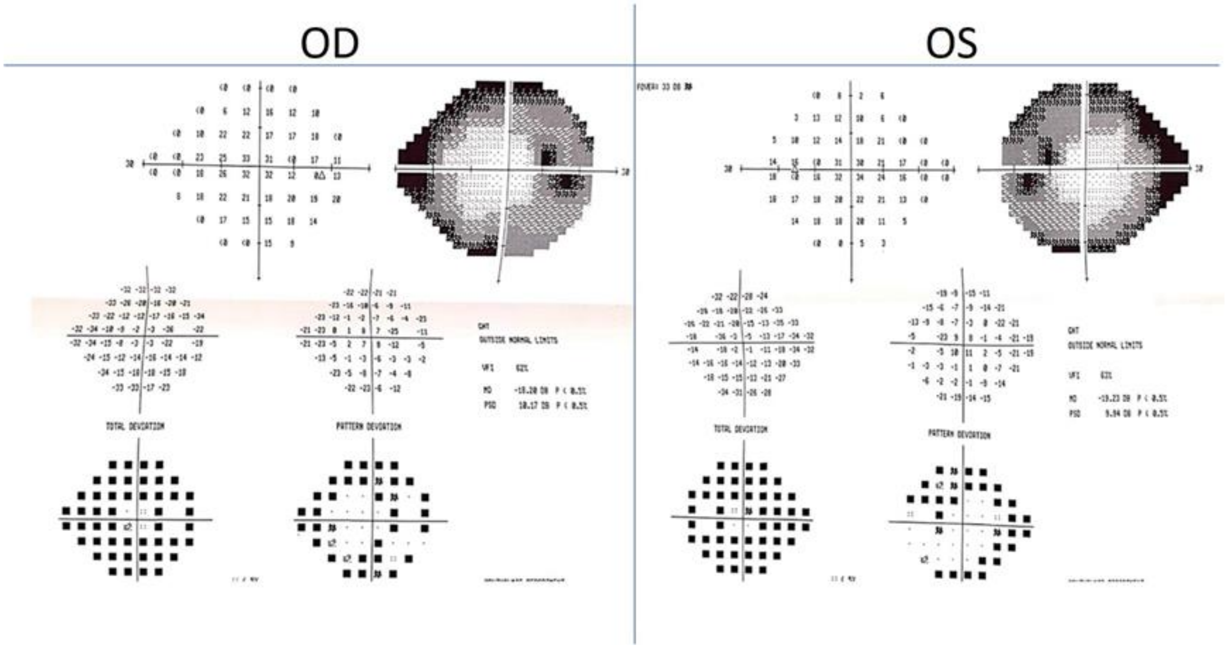

Fig. S2. Single Field Analysis of Visual Fields in Individual III-1 at age 16. OD – right eye, OS – left eye.

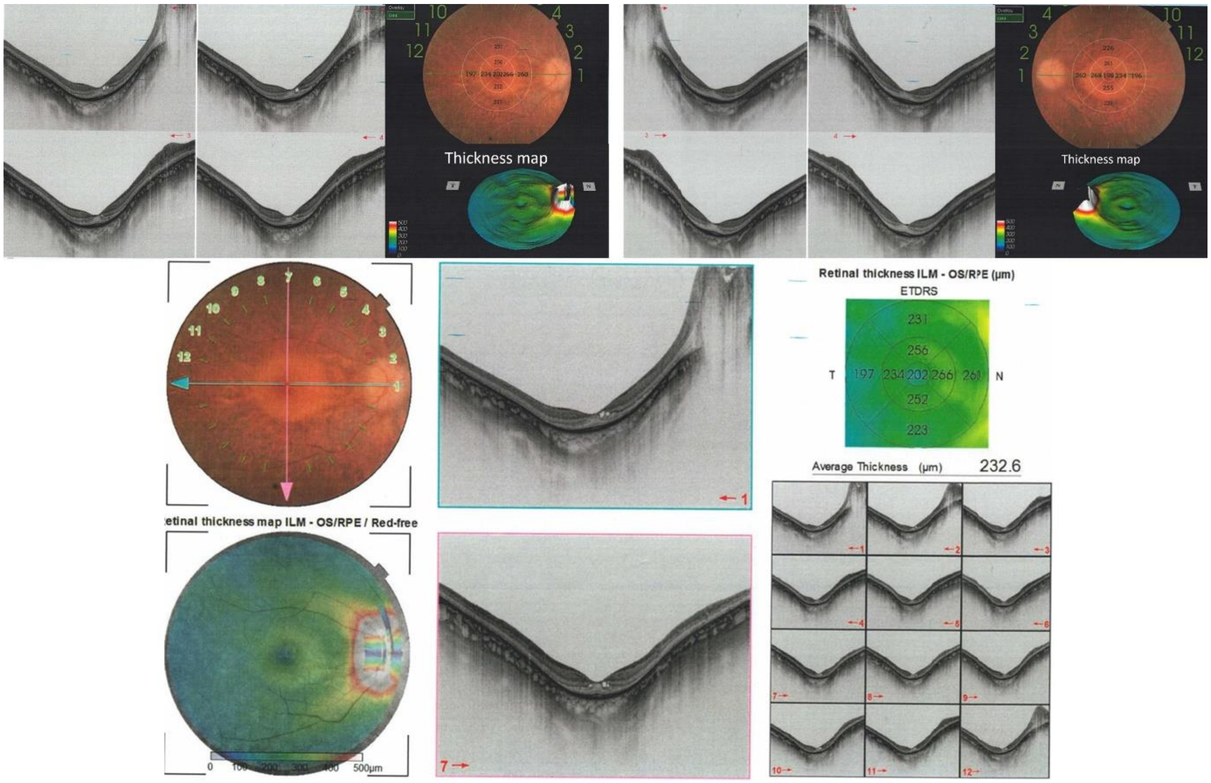

Fig. S3. Individual III-3 at age 26. Fundus photographs of both eyes, SD-OCT imaging, retinal thickness maps (upper part), and a colour photograph of the central retina with its corresponding SD-OCT scan of the right eye (lower part). Please note a pseudo-myopic appearance of the retina: although this individual has only mild myopia, advanced outer retinal thinning related to RP mimics morphology of high myopia, including a pseudo-conus.

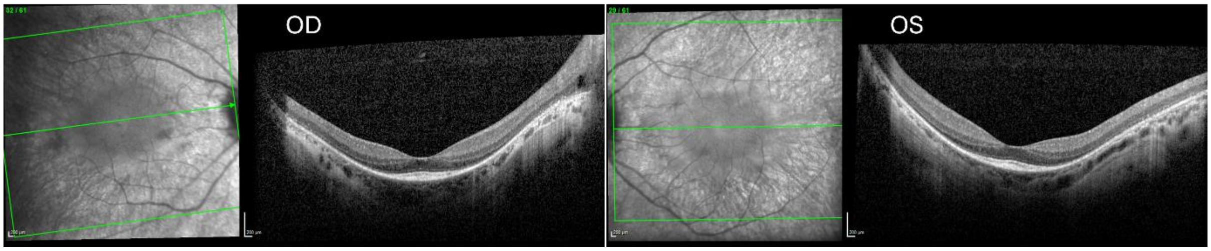

Fig. S4. Individual III-3 at age 28. OCT and fundus images of the right (OD) and left (OS) eyes. Severe retinal atrophy is evident, with almost complete loss of photoreceptors and retinal pigment epithelium layers, except in the central foveal depression.

ATGGTGAGCAAGGGCGAGGAGGATAACATGGCCATCATCAAGGAGTTCATGCGCTTCAAGGT
GCACATGGAGGGCTCCGTGAACGGCCACGAGTTCGAGATCGAGGGCGAGGGCGAGGGCCGC
CCCTACGAGGGCACCCAGACCGCCAAGCTGAAGGTGACCAAGGGTGGCCCCCTGCCCTTCGC
CTGGGACATCCTGTCCCCTCAGTTCATGTACGGCTCCAAGGCCTACGTGAAGCACCCCGCCGA
CATCCCCGACTACTTGAAGCTGTCCTTCCCCGAGGGCTTCAAGTGGGAGCGCGTGATGAACTT
CGAGGACGGCGGCGTGGTGACCGTGACCCAGGACTCCTCCCTGCAGGACGGCGAGTTCATCT
ACAAGGTGAAGCTGCGCGGCACCAACTTCCCCTCCGACGGCCCCGTAATGCAGAAGAAGAC
CATGGGCTGGGAGGCCTCCTCCGAGCGGATGTACCCCGAGGACGGCGCCCTGAAGGGCGAG
ATCAAGCAGAGGCTGAAGCTGAAGGACGGCGGCCACTACGACGCTGAGGTCAAGACCACCT
ACAAGGCCAAGAAGCCCGTGCAGCTGCCCGGCGCCTACAACGTCAACATCAAGTTGGACATC
ACCTCCCACAACGAGGACTACACCATCGTGGAACAGTACGAACGCGCCGAGGGCCGCCACTC
CACCGGCGGCATGGACGAGCTGTACAAGGTTAGCAAAGGTGAAGAACTGTTTACCGGAGTTG
TCCCCATTCTGGTCGAGCTTGATGGCGACGTGAATGGACACAAGTTTAGCGTAAGTGGAGAA
GGAGAAGGAGATGCCACGTATGGAAACTGACCCTGAAGTTCATCTGCACAACTGGGAAATT
GCCTGTGCCTTGGCCGACTTTGGTGACAACCCTCACTTATGGCGTGCAGTGTTTCTCCAGATA
CCCCGATCACATGAAACAGCATGACTTCTTTAAGTCTGCCATGCCAGAAGGGTATGTACAGGA
ACGTACCATCTTCTTCAAAGATGACGGCAACTACAAGACGAGGGCTGAGGTGAAGTTTGAGG
GTGACACACTCGTCAATCGGATTGAGCTGAAAGGCATCGACTTTAAGGAGGATGGGAACATA
CTTGGTCACAAGCTGGAGTACgtaagtgccatgggattgggtggaaacctcgggcaagtacctttcccaaactcttctggggggtcca
tatttggagggaggggagagttctccgttgctctctctttccctcagccctgcctctgatcttcaatgcctttctcacttgt**G**gatgtccttcctgtcacgacag
AACTACAACTCCCATAACGTGTATATCATGGCTGACAAGCAGAAGAATGGCATAAAGGTGAAC
TTCAAAATCCGCCATAATATCGAGGATGGTTCAGTCCAACTCGCAGACCATTACCAGCAGAAC
ACTCCAATTGGCGATGGACCTGTGTTGCTTCCAGACAATCACTACCTGTCAACCCAATCTGCG
CTGAGCAAAGACCCCAATGAGAAACGAGATCACATGGTTCTGCTGGAGTTTGTGACAGCAGC
CGGGATTACCCTCGGCATGGACGAACTGTATAAGTAA

Fig. S5. Design of the Dual Fluorescence Splicing Reporter. The monomeric derivative of DsRed fluorescent protein is shown in red, while the split eGFP (containing 5' and 3' portions) is shown in green. The split eGFP segments are interrupted by the entire 172 bp *PRPF3* wild-type intron 11 (represented in lowercase letters). A capital G in bold within the intron indicates the position of the studied G>A variant.

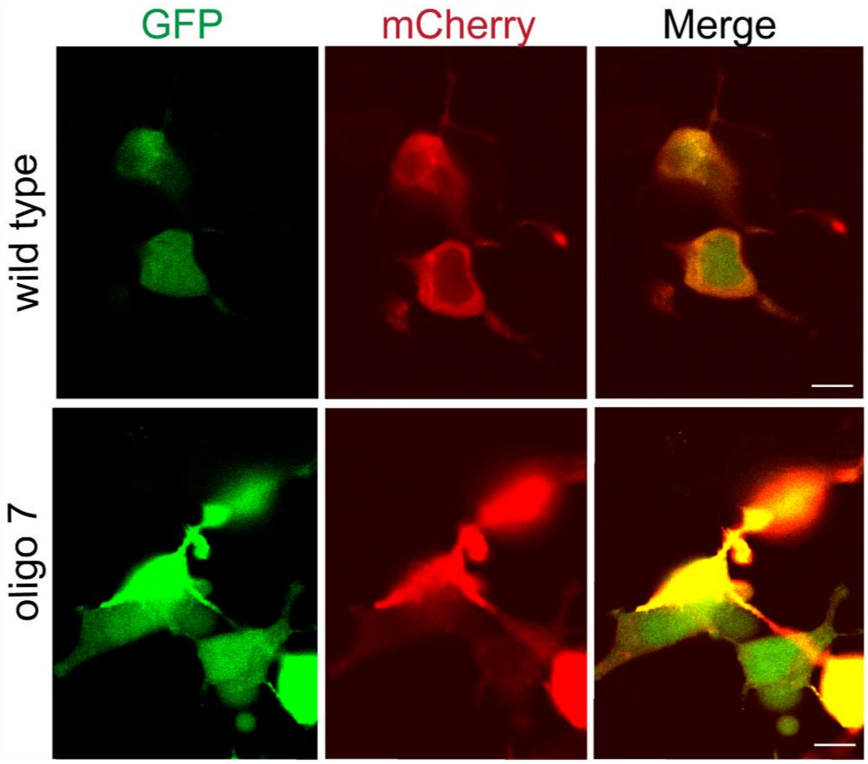

Fig. S6. Confocal microscopy of HEK293T cells transfected with the WT and mutated constructs, alongside AON-7 (750 ng). Merging red and green fluorescence images resulted in yellow signals, indicating co-expression of dual fluorescence in the wild-type samples and splicing restoration of AON-7. Scale bar, 10 µm.
