# Supplementary material for: FACS-based dual fluorescence reporter assay demonstrates efficacy of antisense oligonucleotide therapy of novel PRPF3 intronic splice variant
Source: Mol Ther Nucleic Acids. 2026 May 8;37(2):102949. doi: 10.1016/j.omtn.2026.102949 (PMC13213770; doi:10.1016/j.omtn.2026.102949)
Supplement: Document S2. Article plus supplemental information [file mmc2.pdf]

Molecular Therapy
Nucleic Acids
Brief report

FACS-based dual fluorescence reporter assay demonstrates efficacy of antisense oligonucleotide therapy of novel *PRPF3* intronic splice variant

Vadim Dolgin,[1,2,3] Ekaterina Eremenko,[3,4] Ginat Narkis,[2,3] Masha Mazor-Oring,[1,2] Libe Gradstein,[5,7] and Ohad S. Birk[1,2,3,6,7]

[1]The Morris Kahn Laboratory of Human Genetics, Faculty of Health Sciences, Ben-Gurion University of the Negev, Beer-Sheva, Israel; [2]Genetics Institute, Soroka University Medical Center, Beer-Sheva, Israel; [3]The Shraga Segal Department of Microbiology, Immunology and Genetics, Faculty of Health Sciences, Ben-Gurion University of the Negev, Beer-Sheva, Israel; [4]Zlotowski Neuroscience Center and Regenerative Medicine and Stem Cell Research Center, Ben-Gurion University of the Negev, Beer-Sheva, Israel; [5]Department of Ophthalmology, Soroka Medical Center and Clalit Health Services, Faculty of Health Sciences, Ben-Gurion University of the Negev, Beer-Sheva, Israel; [6]The Danek Gertner Institute of Human Genetics, Sheba Medical Center, Tel Hashomer, Israel

The development of antisense oligonucleotide (AON) therapy for monogenic diseases such as retinitis pigmentosa (RP) is effective but labor-intensive. Rapid, efficient technologies to identify optimal AONs are needed. We present a novel approach focusing on intronic splicing mutations. We identified a novel heterozygous intronic non-canonical splice variant in *PRPF3* causing dominant RP in a four-generation pedigree. Using a Dual Fluorescence Reporter Assay, we transfected HEK293T cells with a reporter plasmid containing either the wildtype intron (resulting in GFP fluorescence due to normal splicing) or the mutant intron (showing no fluorescence due to splicing defects). As an alternative to standard AONs, we used uridine-rich 7 (U7) small nuclear RNA (snRNA) derivatives with optimized Sm protein-binding site and a modified antisense sequence (U7 Sm OPT snRNA) as modulators of pre-mRNA splicing. Eight AONs were designed to target the aberrant *PRPF3* transcript, to restore normal splicing and gene function. To evaluate treatment efficacy, HEK293T cells with the reporter GFP plasmid were co-transfected with U7snRNA cassettes, each containing an incorporated AON sequence. FACS analysis quantified splicing modulation and gene expression rescue, identifying the most effective AON. Our study broadens the genetic understanding of RP, highlighting the significance of personalized medicine in genetic disorder treatment.

INTRODUCTION

Retinitis pigmentosa (RP) is among the most common inherited retinal diseases worldwide, with a prevalence of roughly 1:4000.[1] Comprising a group of genetically heterogeneous disorders that primarily affect the photoreceptor cells in the retina, this condition is marked by the progressive degeneration of photoreceptor cells, leading to significant visual impairment and often resulting in legal blindness in advanced stages. Clinically, RP is characterized by night blindness, followed by the gradual constriction of the visual field, decreased visual acuity, and, in some cases, eventual loss of central vision. While RP can be part of syndromic disorders such as Usher syndrome or Bardet–Biedl syndrome, or a component of complex genetic syndromes, it often occurs in isolation as a non-syndromic condition.[1]

Autosomal dominant RP (adRP) is linked to thousands of mutations across at least 25 genes. Many of these genes, such as *RHO*, *RDS*, and *ROM1*, are predominantly or exclusively expressed in photoreceptor cells, highlighting their critical role in retinal function.[1] In contrast, other genes associated with adRP, such as *PRPF3*, *PRPF8*, and *PRPF31*, are highly conserved and ubiquitously expressed across various tissues being essential components of RNA-splicing complexes.[1] The specific impact of mutations in these ubiquitously expressed genes on retinal photoreceptors, leading to such phenotype-specific manifestations, remains an intriguing and unresolved question in genetic retinal research.

PRPF3 encodes a ubiquitous protein crucial in pre-mRNA splicing. The PRPF3 protein is an essential component of U4/U6 snRNP that binds U6 snRNA.[2,3] In the spliceosome B complex, PRPF3 interacts with PRPF4 and PRPF6, stabilizing U4/U6.U5 tri-snRNP. PRPF3 inactivation compromises spliceosome assembly due to the absence of intact U4/U6.U5 tri-snRNPs.[2,3] Heterozygous *PRPF3* mutations are associated with adRP type 18 (RP18).[4] To date, three

Received 12 January 2025; accepted 5 May 2026;
https://doi.org/10.1016/j.omtn.2026.102949.

[7]These authors contributed equally

Correspondence: Libe Gradstein, Department of Ophthalmology, Soroka Medical Center and Clalit Health Services, Faculty of Health Sciences, Ben-Gurion University of the Negev, Beer-Sheva, Israel.
E-mail: gradl@bgu.ac.il
Correspondence: Ohad S. Birk, Department of Ophthalmology, Soroka Medical Center and Clalit Health Services, Faculty of Health Sciences, Ben-Gurion University of the Negev, Beer-Sheva, Israel.
E-mail: obirk@bgu.ac.il

Published by Elsevier Inc. on behalf of The American Society of Gene and Cell Therapy.
This is an open access article under the CC BY license (http://creativecommons.org/licenses/by/4.0/).

pathogenic or likely pathogenic splice site variants in *PRPF3* have been described, all occurring at the canonical splice sites.[5,6]

Research on inherited retinal diseases has opened many opportunities for the development of molecular therapies over the last several years.[7] Pathogenic intronic and non-canonical splice site mutations resulting in aberrant splicing are excellent candidates for AON-based splicing modulation therapeutic strategies. AONs effectively compete with the spliceosome to recognize splicing motifs. By binding to branch points or cryptic splice sites, AONs create steric hindrances that can shield these regions from being recognized by the spliceosome during splicing. This interference often results in partial or complete restoration of the correctly spliced transcript. However, the effects of AONs are transient due to degradation by endo and exonucleases, suggesting that therapies would require regular reapplication.[8] As an alternative to standard AONs, derivatives of uridine-rich 7 (U7) small nuclear RNA (snRNA) containing optimized Sm protein-binding sites, and a modified antisense sequence (termed U7 Sm OPT snRNA) have proved to be promising modulators of pre-mRNA splicing.[9] Expression of U7 Sm OPT snRNA allows for the continuous nuclear accumulation of therapeutic antisense sequences integrated into a stable small nuclear ribonucleoprotein (snRNP) complex.[10]

To evaluate the effectiveness of AONs in restoring correctly-spliced transcripts, it is common practice to use minigene splice assays *in vitro*. The analysis of splicing products involves reverse transcription (RT)-PCR amplification, which requires multiple experimental steps such as RNA extraction, cDNA synthesis, PCR amplification, gel electrophoresis, and sequencing. These processes can be time-consuming. An alternative time-saving strategy can be utilizing fluorescence reporters.

In this study, we unravel a novel intronic variant in *PRPF3* causing RP in a four-generation pedigree. To address this, we designed AONs to restore normal splicing by targeting the mutated intron. A dual fluorescence reporter was constructed to evaluate and quantify the effects of AONs, using FACS analysis, providing a measurable readout of splicing restoration efficiency. Among the AONs tested, those applied directly to the mutation were found to be more efficient. Our results indicated efficacy of FACS-based Dual Fluorescence Reporter Assay in determining significant restoration of a normal splicing pattern by AONs, highlighting their therapeutic potential.

RESULTS

Patients

Six individuals in four generations of a non-consanguineous Jewish Ashkenazi family presented with classic symptoms of isolated RP, including progressive loss of night vision and impairment of peripheral vision (Figure 1A). The onset was within the first decade of life. Characteristic retinal changes confirmed the diagnosis of RP (Figures 1B and S1–S4). Full-field electroretinogram (ffERG) revealed significant deficiencies in both eyes under photopic (light) and scotopic (dark) conditions, indicating severe and widespread peripheral retinal dysfunction. The rod cells were slightly more affected than the cone cells. These findings demonstrated advanced retinal dysmorphia, consistent with the diagnosis of RP (Table S1).

Identification of the causative variant and RNA studies

Analysis of whole-exome sequencing (individual III-3) and whole-genome sequencing (individual I-1) identified no variants in genes associated with RP, except for an intronic transition variant in *PRPF3*: c.1527-23G>A (NM_004698.4) (Figure 1C). This unique variant was not found in gnomAD, OMIM, HGMD-PRO, ClinVar, Varsome, or dbSNP databases. Segregation analysis within the family (tested members were I-1.2, II-1,2,3,4, III-1,2,3,4,5.6, IV-1,2) confirmed the fully penetrant autosomal dominant pattern of heredity (Figures 1A–1C). The variant was predicted to activate a cryptic splice site in the intron between exons 11 and 12 of *PRPF3*: SpliceAI acceptor gain score = 1 (max); Pangolin splice gain score = 0.88; HSF matrix new acceptor site score – delta 49.28%; MaxEnt new acceptor site score – delta 298.13%; class 5 (max) of splicing effect according to varSEAK tool.

RT-PCR revealed that RNA extracted from the blood of the affected individuals, when compared to wild-type (WT) samples, exhibited an aberrant transcript that included additional 21 base pairs due to intron retention (exonification), which resulted in an in-frame insertion of seven amino acids (p.K509_A510insCPSCHDR; Figures 1C and 1D). This insertion is predicted to impact a highly conserved region of the U4/U6 small nuclear ribonucleoprotein Prp3, one of the multiple proteins comprising the spliceosome (Figures 1E–1G). The proposed effect of the mutant protein may include its altered interaction with U4/U6 snRNA, and/or inability to assemble with other splicing factors (such as SART3; see discussion), leading to a dominant-negative effect, as well as instability and\or mis-localization of the mutant protein. Based on ACMG criteria for variant classification, this transition was classified as pathogenic (PM2, PM4, PP1, PP4, PP3; see also Table S2).

Dual Fluorescence Splicing Reporter minigene

The dual fluorescent reporter is a powerful tool for elucidating the role of specific mutations in splicing impairment and evaluating the efficacy of AONs in restoring disrupted splicing. Our first goal was to confirm the loss of function of the GFP when two of its segments were separated by a mutated intron 11 of *PRPF3* compared to the WT variant. For this purpose, we created a minigene containing a sequence of red fluorescence protein (transfection marker) followed by a split GFP sequence, with *PRPF3* intron 11 positioned between the two GFP segments (Figures 2A and S5). HEK293T cells transfected with the WT and mutated constructs were analyzed using FACS, confocal microscopy, and RT-PCR. Microscopic analysis showed that merging red and green fluorescence images resulted in yellow signals, indicating co-expression of dual fluorescence in the wild-type samples (Figure S6). Interestingly, RT-PCR and FACS analyses revealed a small amount of the normally spliced GFP transcript even in the presence of the mutation (Figures 2B–2D). This could be attributed to simultaneous multiple splicing events influenced by the localization and surrounding genomic

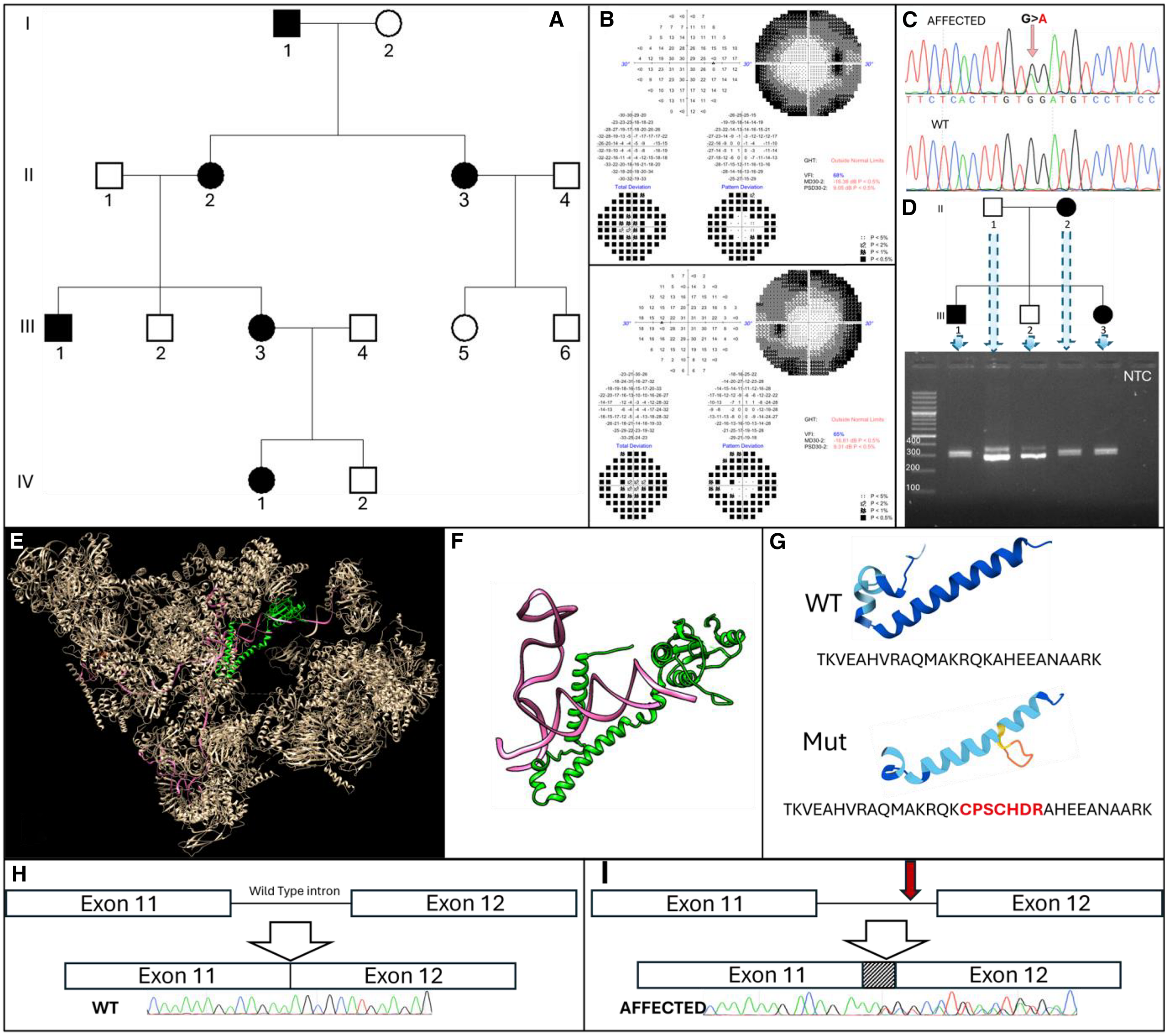

Figure 1. Identification of the *PRPF3* Variant Likely Causing Autosomal Dominant Retinitis Pigmentosa in a Four-Generation Family

(A) The affected kindred: black circles and squares indicate affected individuals. (B) Single Field Analysis of Visual Fields in Individual III-3 at age 23. Upper part – right eye, lower part – left eye. Classic visual field defects associated with RP: peripheral vision loss, evident as darkened areas on the perimetry map, indicating significant deficits in the retinal sensitivity typical for RP. Central vision is relatively preserved, highlighting the progressive nature of the disease. (C) The *PRPF3* Variant Associated with adRP: genomic DNA sequencing chromatogram showing both the wild-type and mutant alleles. The specific nucleotide alteration linked to adRP phenotype is highlighted. (D) Agarose gel electrophoresis of RT-PCR products from cDNA of family members II-1,2 and III-1,2,3. In WT samples, two transcripts are observed: ENST00000467329.5, a processed noncoding transcript at 351 bp, and ENST00000324862.6 (canonical protein coding transcript, NM_004698.4) at 293 bp. The affected sample reveals an additional aberrant transcript at 314 bp. NTC – non-template control. (E,F) In Silico Structural Analysis of the Studied Variant. The Cryo-EM Structure of Human Pre-catalytic Spliceosome (B complex) at 3.8 angstrom resolution. In the green 436-672aa portion of U4/U6 small nuclear ribonucleoprotein, PRPF3 is shown; the pink regions represent the U4 and U6 snRNAs. (G) AlphaFold-predicted 494–519 amino acid alpha helix of PRPF3, including both the wild-type and the mutant, along with the corresponding amino acid sequences. (H,I) Proposed Mechanism of Splicing Alteration: Schematic representation of the proposed mechanism by which the identified mutation (red arrow) leads to altered splicing, specifically resulting in intron retention. Corresponding Sanger sequencing chromatograms of RT-PCR products from wild-type and affected individuals are shown.

context, allowing production of a small amount of correctly spliced transcripts despite the mutation.[11] Together, these findings suggest that the intronic mutation significantly impairs the function of GFP compared to the WT minigene construct.

Further, we investigated whether steric-blocking AONs could be employed to restore the aberrant transcript by either targeting the putative branch points within the mutated intron (AONs 1–6) or by directly addressing the mutation site, thereby preventing the

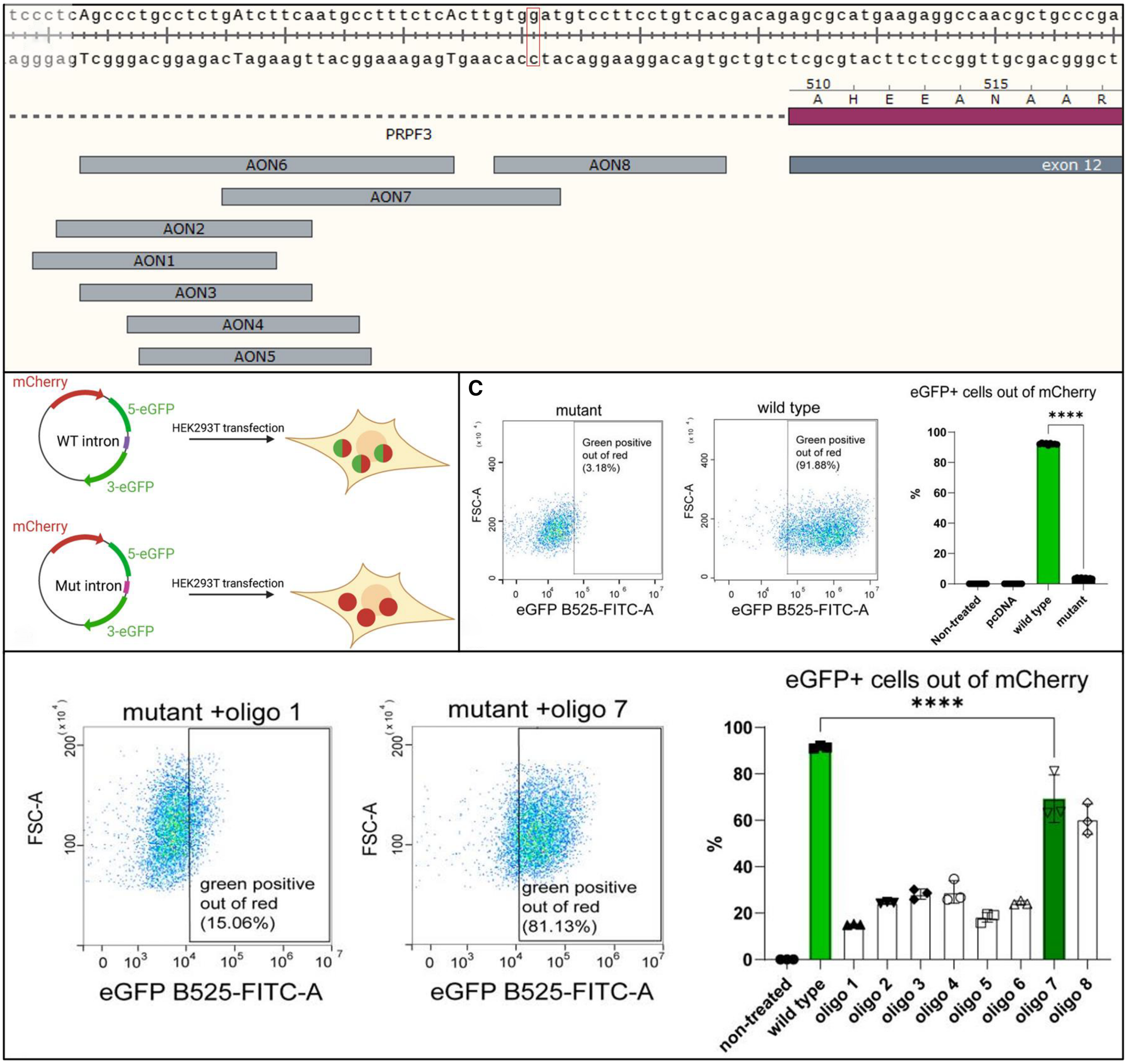

Figure 2. Antisense oligonucleotides (AONs) targeting the *PRPF3* variant

(A) Strategic placement of antisense oligonucleotides relative to the mutation site. The capital intronic A's are putative branch points. The specific nucleotide alteration linked to adRP phenotype is highlighted. (B) Dual Fluorescence Reporter System in HEK293T cells to assess AONs targeting the studied *PRPF3* variant. Schematic representation of the dual fluorescence reporter system used to evaluate the effects of a novel intronic *PRPF3* variant on gene expression in HEK293T cells. Constructs were designed with either a wild-type (WT) or mutant (Mut) intron inserted between the 5′ and 3' ends of the enhanced green fluorescent protein (eGFP) gene. This setup enables assessment of the mutation's impact, as the separation of eGFP exons by the mutated intron 11 of *PRPF3* leads to loss of eGFP function, in contrast to the WT intron. (C) Flow cytometry (FACS) analysis of HEK293T cells transfected with plasmid vectors containing either the WT or mutant intron. The scatterplots show the gating of eGFP-positive cells within the mCherry-positive population, with a marked reduction in green fluorescence observed for cells with the Mut intron construct, indicating impaired eGFP expression. The accompanying bar graph quantifies the percentage of green-positive cells out of red-positive cells for non-transfected cells, cells transfected with pCDNA (control vector), WT intron, and Mut intron constructs, confirming functional impairment induced by the mutant intron. Nine repeats: 3 biological x 3 technical replicates. Analysis using ordinary one-way ANOVA. (D) Assessment of AONs aimed at restoring eGFP expression by targeting the mutant intron. FACS plots show eGFP expression in cells transfected with either WT or mutant intron constructs alongside different AONs, demonstrating partial rescue of eGFP function in the presence of specific AONs. The "mutant" construct contains the mutant intron inserted between the 5′ and 3' ends of the enhanced green fluorescent protein (eGFP) gene. In the transfections, following initial calibrations, 50ng of reporter plasmid was used, along with 500ng of SmOPTs plasmids (molar ratio ∼1:12). Three biological replicates. Analysis using ordinary one-way ANOVA.

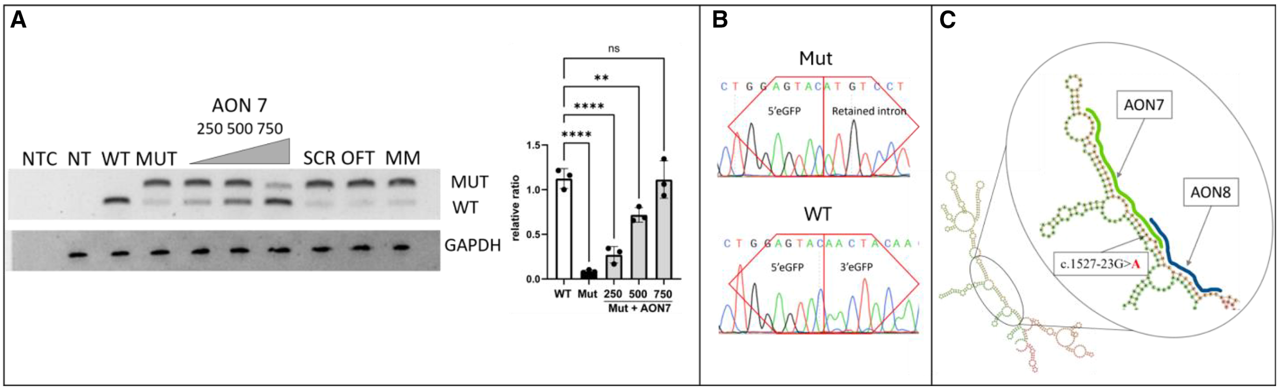

Figure 3. AON-mediated correction of the splicing defect caused by the *PRPF3* variant

(A) Agarose gel electrophoresis analysis of RT-PCR products derived from RNA of HEK293T cells transfected with wild-type and mutant minigene constructs following treatment with a concentration series/varying amounts (250, 500 and 750 ng) of AON 7 and negative controls. Bar graph quantification of the relative amount of normally spliced intron in wild-type (WT), Mutant (Mut), and Mutant samples treated with increasing concentrations of AON7 (250, 500, and 750 ng). Band intensities were quantified using Fiji and normalized to the GAPDH loading control. Data are presented as mean ± SD with individual biological replicates overlaid (n = 3). Statistical significance was determined using a one-way ANOVA with multiple comparisons against the WT control (**$p < 0.01$, ****$p < 0.0001$, ns = not significant). (B) Comparative Sanger sequencing chromatograms of RT-PCR products from untreated mutant and AON-corrected constructs. (C) Computational analysis of RNA secondary structure of the split GFP with *PRPF3* intron between its two portions, showing binding sites for the most effective antisense oligonucleotides. The intronic G>A alteration linked to the adRP phenotype is marked. NTC – non-template control; NT – not treated; SCR – scrambled; OFT – off-target; MM – mismatched; *GAPDH* – glyceraldehyde-3-phosphate dehydrogenase housekeeping gene.

spliceosome from recognizing the cryptic splice site (AONs 7–8) (Figure 3). To achieve this, we set a series of experiments where we co-transfected the reporter plasmid with the mutated intron along with antisense derivatives of U7snRNA with sequences of the above-mentioned AONs introduced in place of the natural 18 bp sequence complementary to the histone pre-mRNA. Our findings reveal that oligonucleotides directed against the mutation site were significantly more effective than those targeting the putative branch-point in restoring normal transcript expression, and this therapeutic effect demonstrated dose-dependent kinetics. (Figure 3).

DISCUSSION

In this study, we delineate a novel intronic variant in *PRPF3* causing RP in a four-generation pedigree. The intronic G>A variant, located 23 bp upstream of exon 12, creates a cryptic acceptor site and intron retention, thereby elongating exon 12 by seven amino acids. The pathogenic variant is located within a highly conserved region (amino acids 416–550) of PRPF3, known to interact with the U4/U6 recycling protein SART3.[12] This interaction is critical, as SART3 plays a pivotal role in mRNA splicing regulation during the spliceosome cycle's recycling phase.[13,14] The disruption caused by the mutation likely contributes to the aberrant splicing patterns seen in affected retinal tissues.

The retina, as one of the most metabolically active tissues, demands an exceptionally high level of splicing activity, as demonstrated by its elevated steady-state levels of small nuclear RNAs and processed pre-mRNAs compared to other tissues.[15] This requirement for increased splicing precision may explain why mutations in ubiquitously expressed splicing factors often present with a narrow ocular phenotype, as observed in RP.

To rescue the aberrant splicing and select the most promising therapeutic to revert the RP phenotype, we designed, created and screened 8 different AONs (each carrying a U7 Sm OPT snRNA cassette with a different AON sequence), targeting the described intronic variant and its putative branch points. Recent investigations have examined the relationship between AONs' length and the efficacy of exon skipping via splice modulation.[16,17] Accordingly, we tested AONs of different lengths (Table S3). In contrast to previous studies of exon skipping, our study focused on targeting cryptic splice sites to remediate intron retention. The selection of AON length was constrained by the spatial proximity of the coding exon to critical regulatory elements, including the polypyrimidine tract, branchpoint sequence, and splice acceptor site. In parallel, several negative control (scramble, mismatch, of target) oligos were tested to ensure that observed effects are due to the specific AON sequence targeting the disease-related RNA, not off-target effects or nonspecific interactions.

The rationale for using the U7 snRNA scaffold in antisense therapy is primarily based on its natural role in modifying histone mRNA and its ability to be engineered to target specific RNA sequences. U7 snRNA is a small nuclear RNA involved in histone pre-mRNA processing, and it can be modified to carry antisense sequences that guide the correction or modulation of abnormal splicing, degradation, or expression of target RNAs. By incorporating therapeutic antisense sequences into the U7 snRNA scaffold, one can make use of the natural properties of snRNA molecules to improve treatment effectiveness. The stability of snRNA helps the therapeutic agents

remain intact inside the cell, while their ability to be efficiently delivered ensures they reach their target locations. Since snRNAs are naturally transported into the nucleus, this facilitates the precise targeting of disease-related RNA transcripts, such as mutant pre-mRNAs. This allows for the modulation of splicing patterns, reduction of toxic RNA buildup, or restoration of normal gene expression. Overall, the U7 snRNA scaffold provides a versatile and effective platform for antisense therapy, especially in correcting abnormal RNA processing associated with various genetic disorders.To facilitate quantification of the splicing products via FACS we used a modified dual fluorescence reporter[17] consisting of red fluorescence signal as a transfection marker, and eGFP split by WT/mutated *PRPF3* intron 11 sequence; when the WT intron is spliced correctly, GFP is detected and serves as a standard for comparison when AONs are applied to the mutant intron. Thus, the dual fluorescence reporter was constructed to evaluate and quantify the effects of AONs, providing a measurable readout of splicing restoration efficiency. Of the AONs evaluated, those designed to target the mutation site directly exhibited greater therapeutic efficacy. This improved performance likely results from steric hindrance that prevents spliceosome recognition of the cryptic splice site upon AON binding. Our results indicated significant restoration of normal splicing pattern in the presence of AONs, highlighting their potential as a therapeutic strategy. This reinforces the therapeutic promise of AONs, not only for *PRPF3*-associated adRP but potentially for a broader spectrum of splicing-related disorders. As important, our findings demonstrate the efficacy of FACS-based dual fluorescence reporter assay in determining efficacy of AON therapy. Future studies will focus on refining AON delivery and examining long-term impacts on retinal function to validate this approach further.

Importantly, we acknowledge the limitations of the current work, primarily its reliance on an *in vitro* reporter system. While our data provide strong proof-of-concept for AON-mediated splice-switching at the transcript level, further functional validation, including demonstrating endogenous Prp3 protein rescue and phenotypic improvement in relevant *in vivo* models, will be necessary to fully support the therapeutic relevance of our findings.

Inherited retinal degenerations are associated with more than 300 causative genes, with *PRPF3* alone harboring dozens of pathogenic variants.[1] Although only a minority of these mutations directly affect splicing, there remains a critical need for effective therapeutic strategies for splicing-specific interventions.[1] While the development of mutation-independent therapies is gaining momentum, the substantial biocomplexity of these disorders continues to present major challenges and contributes to a significant healthcare burden for patients awaiting treatment. Addressing this genetic and mechanistic heterogeneity will likely require a diverse portfolio of complementary approaches. As demonstrated here, the dual-fluorescence assay represents one such tool with potential utility in this context.

In conclusion, we demonstrate the first intronic non-canonical splice mutation in *PRPF3* causing adRP. Furthermore, effectively employing the Dual Fluorescence Splicing Reporter, we highlight the efficacy of this assay combined with FACS analysis in facilitating quantification of splicing in general, and most notably when studying a specific variant to design and select effective AONs. Finally, we further highlight this technology as a powerful, time-saving tool in developing antisense-oligo splice-switching therapy.

MATERIALS AND METHODS

Ethics statement

All procedures were carried out following written informed consent, under the principles outlined in the Declaration of Helsinki (Soroka Medical Center IRB approval No. 5071G).

Clinical phenotyping

A senior geneticist and ophthalmologist examined affected individuals. Patients underwent thorough ophthalmic examination, including ERG.

Genomic DNA extraction from whole blood or saliva

Blood or saliva samples were obtained following written informed consent from all individuals studied or their legal guardians. Blood samples (3–10 mL) were collected in BD EDTA tubes, and total genomic DNA was extracted using E.Z.N.A. Blood DNA Kit. Saliva samples were collected in OG-500 Oragene Saliva DNA Collection Kit (Oragene, Ottawa, Ontario, Canada), and genomic DNA was extracted per the manufacturer's instructions.

DNA sequencing and data analysis

Sequencing, read alignment, variant calling, and annotation were performed by Macrogen Europe B.V. (Amsterdam, Netherlands). Whole exome sequencing (WES) was performed on the Illumina NovaSeq6000 machine using Agilent SureSelect Exome V7 post-capture library construction, 150 pair end protocol. Whole genome sequencing (WGS) was performed on the Illumina NovaSeq6000 machine using TruSeq PCR-free (350 bp insert) protocol ($\sim$110 Gb raw data).

Sequencing data were analyzed through the use of Ingenuity Variant Analysis software (https://www.qiagenbioinformatics.com/products/ingenuity-variant-analysis) from QIAGEN, Inc., and the Varista software,[18] excluding variants observed with an allele frequency $\geq$1% in the 1000 Genomes Project[19] or the Allele Frequency Community,[20] or variants appearing in a homozygous state in our in-house WES database of 900 controls. Variants were then further sifted based on their being predicted deleterious (as listed in HGMD or ClinVar), previously classified as disease-associated (pathogenic or likely pathogenic) according to computed ACMG guidelines classification, or being associated with loss of function by causing frameshift, in-frame indel, start/stop codon change, missense or splice site loss up to 5 bases into intron (as predicted by MaxEntScan[21]). Deep intronic variants were analyzed using SpliceAI,[22] VarSeak (https://varseak.bio), and Human Splice Finder[23] tools. In silico protein prediction was processed via AlphaFold Server,[24] based on high resolution Cryo-EM structure of the human pre-catalytic spliceosome[25] with

corresponding PDB structure (https://doi.org/10.2210/pdb6AHD/pdb). RNA structures were analyzed using the RNAfold web server.[26]

Segregation analysis

The *PRPF3* intronic variant was further assayed through Sanger sequencing using forward and reverse primers given in Table S4. For co-segregation we also applied restriction fragment-length polymorphism (RFLP) using the restriction endonuclease BtsCI (New England Biolabs, Ipswich, MA, USA). Primers were designed using Primer-Blast.

RNA isolation and cDNA synthesis

For RNA isolation, blood samples were collected into Tempus (Applied Biosystems, Foster City, CA) tubes (3 mL blood per tube) according to the manufacturer's instructions. RNA was extracted using the Direct-zol RNA MiniPrep Plus kit (Zymo Research) as indicated in the manufacturer's instructions. For cDNA synthesis, 0.5 μg of DNaseI-treated total RNA was used as the initial input for the reaction, which was performed using the qPCRBIO cDNA Synthesis Kit (PCR Biosystems, London, UK) according to the manufacturer's instructions.

RT-PCR analysis

Whole RNA splicing profiles were analyzed by RT-PCR using agarose gel electrophoresis, Sanger sequencing, and/or high-resolution capillary electrophoresis in the QIAxcel Advanced system. cDNA derived from blood samples RNA or lysed HEK 293 T cells was used as a template for PCR (primer sequences in Table S4). Following electrophoresis, distinct PCR products were extracted from a 2% agarose gel using a sterile scalpel blade and purified with the QIAquick gel extraction kit (Qiagen), after which the eluates underwent Sanger sequencing. Chromatograms were parsed with Poly Peak Parser[27] (http://yosttools.genetics.utah.edu/PolyPeakParser/).

Dual Fluorescence Splicing Reporter construction

The insert - 1615 bp nucleotide sequence (Figure S5) consisting of a monomeric derivative of dsRed fluorescent protein[28] and following split eGFP interceded by the entire 172bp *PRPF3* WT intron 11 sequence (NM_004698.4), was synthesized by Twist Bioscience HQ (CA 94080, USA). The construct was further cloned independently into two different vectors.

1. For introducing the insert into pcDNA3.1(−) vector, Gibbson assembly was utilized. The vector was PCR-linearized with primers FMv2_vec (see Table S4); the insert was PCR-modified with primers FMv2_ins. DH5a-competent Escherichia coli were transformed with the generated plasmid, and positive clones were verified through Sanger sequencing. The G to A mutation was introduced by Q5 site-directed mutagenesis with primers MUT-FMv2_*PRPF3.*
2. A double digestion-ligation approach was utilized to introduce the insert into the pMP71Gpre vector,[29] which was generously shared by Dr. Eremenko. For this purpose, the insert was PCR-modified, column-purified, and digested with NOTI and EcoRI enzymes (NEB). pMP71Gpre vector was digested with the same enzymes, separated from its GFP insert by excision from agarose gel, and purified. The backbone and insert were ligated using T4 DNA ligase (NEB). The G to A mutation was introduced as described above.

Design of antisense oligonucleotides (AONs)

Utilizing the map of splicing elements in *PRPF3* intron 11, as predicted by various bioinformatics tools (VarSeak, Human Splice Finder, ESEfinder 3.0, RNA structure), we picked eight sequences (Table S3) anticipated to target either the branchpoint involved in mutant splicing or the mutation site itself (Figure 3C). Using these sequences, we substituted the 18 bp sequence complementary to the histone pre-mRNA (AAGTGTTACAGCTCTTTT) within the mouse U7-snRNA gene (see below).

Design of U7snRNA vectors

Fourteen 445 bp fragments of the mouse U7-snRNA gene, which include promoter and terminator sequences, were synthesized by Twist Bioscience HQ (CA 94080, USA) with the following modifications: 5′-ttaaGCTAGC and 3′-GAATTCaatt tails, allowing NheI and EcoRI subsequent cloning; The U7-specific Sm binding site (AATTTGTCTAG) was replaced with the consensus Sm binding site (AATTTTTGGAG), thereby integrating the modified snRNA into the snRNP complex that targets the spliceosome; The 18 bp sequence complementary to the histone pre-mRNA (AAGTGTTACAGCTCTTTT) was substituted with fourteen antisense sequences, each targeting the region of the studied pre-mRNA (see above) or serving as negative control.

The fragments were introduced into pcDNA3.1(−) vector by a double (NheI and EcoRI) digestion-ligation approach. The appropriate introduction of all fragments was validated by Sanger sequencing.

U7snRNAs studies in HEK293T cells

HEK293T cells (American Type Culture Collection no. CRL-3216) were cultured in DMEM supplemented with 10% fetal bovine serum, 1% penicillin-streptomycin, and 2 mM L-glutamine at 37°C with 5% CO_2. Transfection and co-transfection experiments were carried out on 70% confluent cells in 6 or 12-well plates using PolyJet transfection reagent (SignaGen Laboratories, Rockville, MD, USA) according to the manufacturer's protocol. In the transfections, following initial calibrations, 50ng of reporter plasmid was used, along with 500ng of SmOPTs plasmids (molar ratio ∼1:12). For AON 7, further optimization studies were done using 3 different AON concentrations (Figure 3 and its legend). Forty-eight hours post-transfection, the cells were harvested for RT-PCR and/or Flow cytometry (FCM) analysis. Amplification of U7snRNA short and long fragments (primers u7ext and u7int, Table S4) was used as AON-carrying vector transfection control.

Flow cytometry (FCM)

HEK293T cells were plated in 12-well plates with DMEM (Dulbecco's Modified Eagle Medium) containing 10% fetal bovine serum (FBS) and 1% penicillin-streptomycin (Pen-Strep). The cells were

incubated for 16 h at 37°C in a humidified atmosphere with 5% CO_2. Subsequently, they were transfected with the appropriate plasmid DNA using the PolyJet transfection reagent, following the manufacturer's instructions. Forty-eight hours post-transfection, cells were harvested by trypsinization, rinsed twice with phosphate-buffered saline (PBS), and stained with eBioscience Fixable Viability Dye eFluor 780 (Invitrogen) in FACS buffer (PBS containing 2% FBS) for 10 min at room temperature, shielded from light. After staining, the cells were washed twice with FACS buffer to remove residual dye. Analysis was conducted using a Cytoflex LX flow cytometer, with settings optimized to detect eFluor 780, EGFP, and dsRed fluorescence. Data analysis was performed with Cytexpert software to evaluate cell viability and to measure EGFP and dsRed fluorescence. EGFP fluorescence intensity was measured by flow cytometry and expressed as median fluorescence intensity (MFI) or percentage of GFP positive cells. Statistical analysis was performed using one-way ANOVA followed by post hoc test to compare treatment groups. A p-value <0.05 was considered statistically significant.

DATA AND CODE AVAILABILITY

The data generated and analyzed in the current study are available from the corresponding author upon reasonable request.

ACKNOWLEDGMENTS

This work was supported by the Israel Science Foundation (grant 2463/23 to OSB) and the Sheba-Kadar prize (OSB). We extend our sincere gratitude to the family members who participated in this study. We are deeply grateful for their trust and cooperation throughout the research process.

AUTHOR CONTRIBUTIONS

VD – conceptualization, methodology, investigation, data curation, formal analysis, writing – original draft preparation; EE – methodology, validation; GN – writing – review and editing, supervision; MMO – clinical investigation; LG – clinical investigation; OSB – resources, writing – review and editing, supervision, funding acquisition, and project administration.

DECLARATION OF INTERESTS

The authors declare that there are no conflicts of interest regarding the publication of this paper.

SUPPLEMENTAL INFORMATION

Supplemental information can be found online at https://doi.org/10.1016/j.omtn.2026.102949.

REFERENCES

1. Fahim, A.T., Daiger, S.P., and Weleber, R.G. (2023). Nonsyndromic Retinitis Pigmentosa Overview. In GeneReviews® [Internet], M.P. Adam, S. Bick, G.M. Mirzaa, R.A. Pagon, S.E. Wallace, and A. Amemiya, eds. (University of Washington, Seattle), pp. 1993–2026, https://www.ncbi.nlm.nih.gov/books/NBK1417/.

2. Liu, S., Rauhut, R., Vornlocher, H.-P., and Lührmann, R. (2006). The network of protein-protein interactions within the human U4/U6.U5 tri-snRNP. RNA *12*, 1418–1430. https://doi.org/10.1261/rna.55406.

3. Liu, S., Mozaffari-Jovin, S., Wollenhaupt, J., Santos, K.F., Theuser, M., Dunin-Horkawicz, S., Fabrizio, P., Bujnicki, J.M., Lührmann, R., and Wahl, M.C. (2015). A composite double-/single-stranded RNA-binding region in protein Prp3 supports tri-snRNP stability and splicing. eLife *4*, e07320. https://doi.org/10.7554/eLife.07320.

4. Chakarova, C.F., Hims, M.M., Bolz, H., Abu-Safieh, L., Patel, R.J., Papaioannou, M.G., Inglehearn, C.F., Keen, T.J., Willis, C., Moore, A.T., et al. (2002). Mutations in HPRP3, a third member of pre-mRNA splicing factor genes, implicated in autosomal dominant retinitis pigmentosa. Hum. Mol. Genet. *11*, 87–92. https://doi.org/10.1093/hmg/11.1.87.

5. Wang, J., Xiao, X., Li, S., Jiang, H., Sun, W., Wang, P., and Zhang, Q. (2022). Landscape of pathogenic variants in six pre-mRNA processing factor genes for retinitis pigmentosa based on large in-house data sets and database comparisons. Acta Ophthalmol. *100*, e1412–e1425. https://doi.org/10.1111/aos.15104.

6. Koyanagi, Y., Akiyama, M., Nishiguchi, K.M., Momozawa, Y., Kamatani, Y., Takata, S., Inai, C., Iwasaki, Y., Kumano, M., Murakami, Y., et al. (2019). Genetic characteristics of retinitis pigmentosa in 1204 Japanese patients. J. Med. Genet. *56*, 662–670. https://doi.org/10.1136/jmedgenet-2018-105691.

7. Tomkiewicz, T.Z., Suárez-Herrera, N., Cremers, F.P.M., Collin, R.W.J., and Garanto, A. (2021). Antisense Oligonucleotide-Based Rescue of Aberrant Splicing Defects Caused by 15 Pathogenic Variants in ABCA4. Int. J. Mol. Sci. *22*, 4621. https://doi.org/10.3390/ijms22094621.

8. Dominski, Z., and Kole, R. (1993). Restoration of correct splicing in thalassemic pre-mRNA by antisense oligonucleotides. Proc. Natl. Acad. Sci. USA *90*, 8673–8677. https://doi.org/10.1073/pnas.90.18.8673.

9. Gadgil, A., and Raczyńska, K.D. (2021). U7 snRNA: A tool for gene therapy. J. Gene Med. *23*, e3321. https://doi.org/10.1002/jgm.3321.

10. Lu, Q.-L., Yokota, T., Takeda, S., Garcia, L., Muntoni, F., and Partridge, T. (2011). The status of exon skipping as a therapeutic approach to duchenne muscular dystrophy. Mol. Ther. *19*, 9–15. https://doi.org/10.1038/mt.2010.219.

11. Ren, P., Lu, L., Cai, S., Chen, J., Lin, W., and Han, F. (2021). Alternative Splicing: A New Cause and Potential Therapeutic Target in Autoimmune Disease. Front. Immunol. *12*, 713540. https://doi.org/10.3389/fimmu.2021.713540.

12. Song, E.J., Werner, S.L., Neubauer, J., Stegmeier, F., Aspden, J., Rio, D., Harper, J.W., Elledge, S.J., Kirschner, M.W., and Rape, M. (2010). The Prp19 complex and the Usp4Sart3 deubiquitinating enzyme control reversible ubiquitination at the spliceosome. Genes Dev. *24*, 1434–1447. https://doi.org/10.1101/gad.1925010.

13. Didychuk, A.L., Butcher, S.E., and Brow, D.A. (2018). The life of U6 small nuclear RNA, from cradle to grave. RNA *24*, 437–460. https://doi.org/10.1261/rna.065136.117.

14. Black, C.S., Whelan, T.A., Garside, E.L., MacMillan, A.M., Fast, N.M., and Rader, S.D. (2023). Spliceosome assembly and regulation: insights from analysis of highly reduced spliceosomes. RNA *29*, 531–550. https://doi.org/10.1261/rna.079273.122.

15. Tanackovic, G., Ransijn, A., Ayuso, C., Harper, S., Berson, E.L., and Rivolta, C. (2011). A missense mutation in PRPF6 causes impairment of pre-mRNA splicing and autosomal-dominant retinitis pigmentosa. Am. J. Hum. Genet. *88*, 643–649. https://doi.org/10.1016/j.ajhg.2011.04.008.

16. Fukushima, S., Farea, M., Maeta, K., Rani, A.Q.M., Fujioka, K., Nishio, H., and Matsuo, M. (2020). Dual Fluorescence Splicing Reporter Minigene Identifies an Antisense Oligonucleotide to Skip Exon v8 of the CD44 Gene. Int. J. Mol. Sci. *21*, 9136. https://doi.org/10.3390/ijms21239136.

17. Shimo, T., Ueda, O., and Yamamoto, S. (2024). Design and evaluation of antisense sequence length for modified mouse U7 small nuclear RNA to induce efficient pre-messenger RNA splicing modulation in vitro. PLoS One *19*, e0305012. https://doi.org/10.1371/journal.pone.0305012.

18. Hadar, N., Dolgin, V., Oustinov, K., Yogev, Y., Poleg, T., Safran, A., Freund, O., Agam, N., Jean, M.M., Proskorovski-Ohayon, R., et al. (2024). VARista: a free web platform for streamlined whole-genome variant analysis across T2T, hg38, and hg19. Hum. Genet. *143*, 695–701. https://doi.org/10.1007/s00439-024-02671-4.

19. 1000 Genomes Project Consortium, Auton, A., Brooks, L.D., Durbin, R.M., Garrison, E.P., Kang, H.M., Korbel, J.O., Marchini, J.L., McCarthy, S., McVean, G.A., and Abecasis, G.R. (2015). A global reference for human genetic variation. Nature *526*, 68–74. https://doi.org/10.1038/nature15393.

20. Gonzalez-Galarza, F.F., Christmas, S., Middleton, D., and Jones, A.R. (2011). Allele frequency net: a database and online repository for immune gene frequencies in worldwide populations. Nucleic Acids Res. *39*, D913–D919. https://doi.org/10.1093/nar/gkq1128.

21. Yeo, G., and Burge, C.B. (2004). Maximum entropy modeling of short sequence motifs with applications to RNA splicing signals. J. Comput. Biol. *11*, 377–394. https://doi.org/10.1089/1066527041410418.

22. Jaganathan, K., Kyriazopoulou Panagiotopoulou, S., McRae, J.F., Darbandi, S.F., Knowles, D., Li, Y.I., Kosmicki, J.A., Arbelaez, J., Cui, W., Schwartz, G.B., et al. (2019). Predicting Splicing from Primary Sequence with Deep Learning. Cell *176*, 535–548.e24. https://doi.org/10.1016/j.cell.2018.12.015.

23. Desmet, F.-O., Hamroun, D., Lalande, M., Collod-Béroud, G., Claustres, M., and Béroud, C. (2009). Human Splicing Finder: an online bioinformatics tool to predict splicing signals. Nucleic Acids Res. *37*, e67. https://doi.org/10.1093/nar/gkp215.

24. Abramson, J., Adler, J., Dunger, J., Evans, R., Green, T., Pritzel, A., Ronneberger, O., Willmore, L., Ballard, A.J., Bambrick, J., et al. (2024). Accurate structure prediction of biomolecular interactions with AlphaFold 3. Nature *630*, 493–500. https://doi.org/10.1038/s41586-024-07487-w.

25. Zhan, X., Yan, C., Zhang, X., Lei, J., and Shi, Y. (2018). Structures of the human pre-catalytic spliceosome and its precursor spliceosome. Cell Res. *28*, 1129–1140. https://doi.org/10.1038/s41422-018-0094-7.

26. Hill, J.T., Demarest, B.L., Bisgrove, B.W., Su, Y.-C., Smith, M., and Yost, H.J. (2014). Poly peak parser: Method and software for identification of unknown indels using sanger sequencing of polymerase chain reaction products. Dev. Dyn. *243*, 1632–1636. https://doi.org/10.1002/dvdy.24183.

27. Shaner, N.C., Campbell, R.E., Steinbach, P.A., Giepmans, B.N.G., Palmer, A.E., and Tsien, R.Y. (2004). Improved monomeric red, orange and yellow fluorescent proteins derived from Discosoma sp. red fluorescent protein. Nat. Biotechnol. *22*, 1567–1572. https://doi.org/10.1038/nbt1037.

28. Gruber, A.R., Lorenz, R., Bernhart, S.H., Neuböck, R., and Hofacker, I.L. (2008). The Vienna RNA Websuite. Nucleic Acids Res. *36*, W70–W74. https://doi.org/10.1093/nar/gkn188.

29. Engels, B., Cam, H., Schüler, T., Indraccolo, S., Gladow, M., Baum, C., Blankenstein, T., and Uckert, W. (2003). Retroviral Vectors for High-Level Transgene Expression in T Lymphocytes. Hum. Gene Ther. *14*, 1155–1168. https://doi.org/10.1089/104303403322167993.

OMTN, Volume *37*

Supplemental information

FACS-based dual fluorescence reporter assay demonstrates efficacy of antisense oligonucleotide therapy of novel *PRPF3* intronic splice variant

Vadim Dolgin, Ekaterina Eremenko, Ginat Narkis, Masha Mazor-Oring, Libe Gradstein, and Ohad S. Birk

Supplemental Figures and Tables

Table S1. Full-field ERG results in Individual III-1 at age 18.

		OD	OS	Reference
Light-adapted 3.0 ERG	b-wave: amplitude, µv<br>b-wave: latency, msec	14<br>32	17<br>32	Min 85<br>Max 32
Light-adapted 3.0 flicker	amplitude, µv<br>latency, msec	-<br>-	-<br>-	Min 65<br>Max 30
Dark-adapted blue light ERG	Rod response, µv	8	-	Min 60
Dark-adapted red light ERG	Cone response, µv	12	10	Min 50
Dark-adapted 3.0 ERG	a-wave, µv<br>a-wave, µv	4<br>6	3<br>6	Min 120<br>Min 280
Dark-adapted 30 ERG	a-wave, µv<br>a-wave, µv	2<br>7	4<br>6	Min 210<br>Min 320

Table S2. ACMG criteria for variant *PRPF3*: c.1527-23G>A (NM_004698.4) classification.

Criteria	PM2 sup	PM4 sup	PP1 strong	PP4 sup	PP3 strong
Notes	Allele frequency is extremely low	Protein length changes resulting from in-frame insertion in a non-repeat region	Cosegregation with disease in multiple affected family members in a gene definitively known to cause the disease	Patient's phenotype or family history is highly specific for a disease with a single genetic etiology.	Computational prediction tools unanimously support a deleterious effect on the gene

Table S3. AON sequences.

AON 1	AGAUCAGAGGCAGGGCUGAGG
AON 2	UGAAGAUCAGAGGCAGGGCUGA
AON 3	UGAAGAUCAGAGGCAGGGCU
AON 4	GCAUUGAAGAUCAGAGGCAG
AON 5	GGCAUUGAAGAUCAGAGGCA
AON 6	UGAGAAAGGCAUUGAAGAUCAGAGGCAGGGCU
AON 7	AUC**U**ACAAGUGAGAAAGGCAUUGAAGAUC
AON 8	GUGACAGGAAGGACAUC**U**AC
SCRAMBLED1	AUGGCAUUGAAGCUACAAGUGAGAAAAUC
SCRAMBLED2	AUCUACAAGUGAGAAAAGUUACGGAGAUC
OFTARGET1	GCAUCAUCAUUACUACUACG
OFTARGET2	GAUACGAUACAGACAUAUAC
MISMATCH1	AUCCACAAUUGAAAAAGUCAUUGAAGAUC
MISMATCH2	AUCUUCAAGUGAGACAGGCACUGAAUAUC

Table S4. Primers used.

Primer name	Sequence from 5' to 3'
g*PRPF3*-F	GGTGGAAACCTCGGGCAAGTA
g*PRPF3*-R	AGTAGGAACTGGTGTTAGGTATGCT
cDNA-*PRPF3*-F	CTCCAGAACCCAAAGTGAGAATTTC
cDNA-*PRPF3*-R	GTACAGTTGCCCAGCATTGG
FMv2_vec-F	TGTATAAGTAATTTAAACCGCTGATCAGCCTCG
FMv2_vec-R	CTCACCATCAGCTTGGGTCTCCCTATAGTGAG
FMv2_ins-F	CCAAGCTGATGGTGAGCAAGGGCGAG
FMv2_ins-R	CAGCGGTTTAAATTACTTATACAGTTCGTCCATGCCGAG
MUT-FMv2_*PRPF3*-F	TCTCACTTGTaGATGTCCTTC
MUT-FMv2_*PRPF3*-R	AAGGCATTGAAGATCAGAG
NOT1-FMpMP-F	aaaaagcggcCGCCACCATGGTGAGCAAG
EcoRI-FMpMP-R	aaaaaagaatTCGAGTTACTTATACAGTTCGTCCATGCCG
u7ext-F	CTGGCTAGCTAACAACATAGGAGCTG
u7ext-R	GAAACCAGAGAAGGATCAAAGCCC
u7int-F	CTACAGACGCACTTCCGCAA
u7int-R	GAAGTCAGAAAACCTGCTCCAAAAATT
GAPDH-F	TCAACAGCGACACCCACTCC
GAPDH-R	AGCCAAATTCGTTGTCATACCAGG
GFP-F5	AGTTTGAGGGTGACACACTC
GFP-R3	CCATGATATACACGTTATGGGAGTTGTAGTT

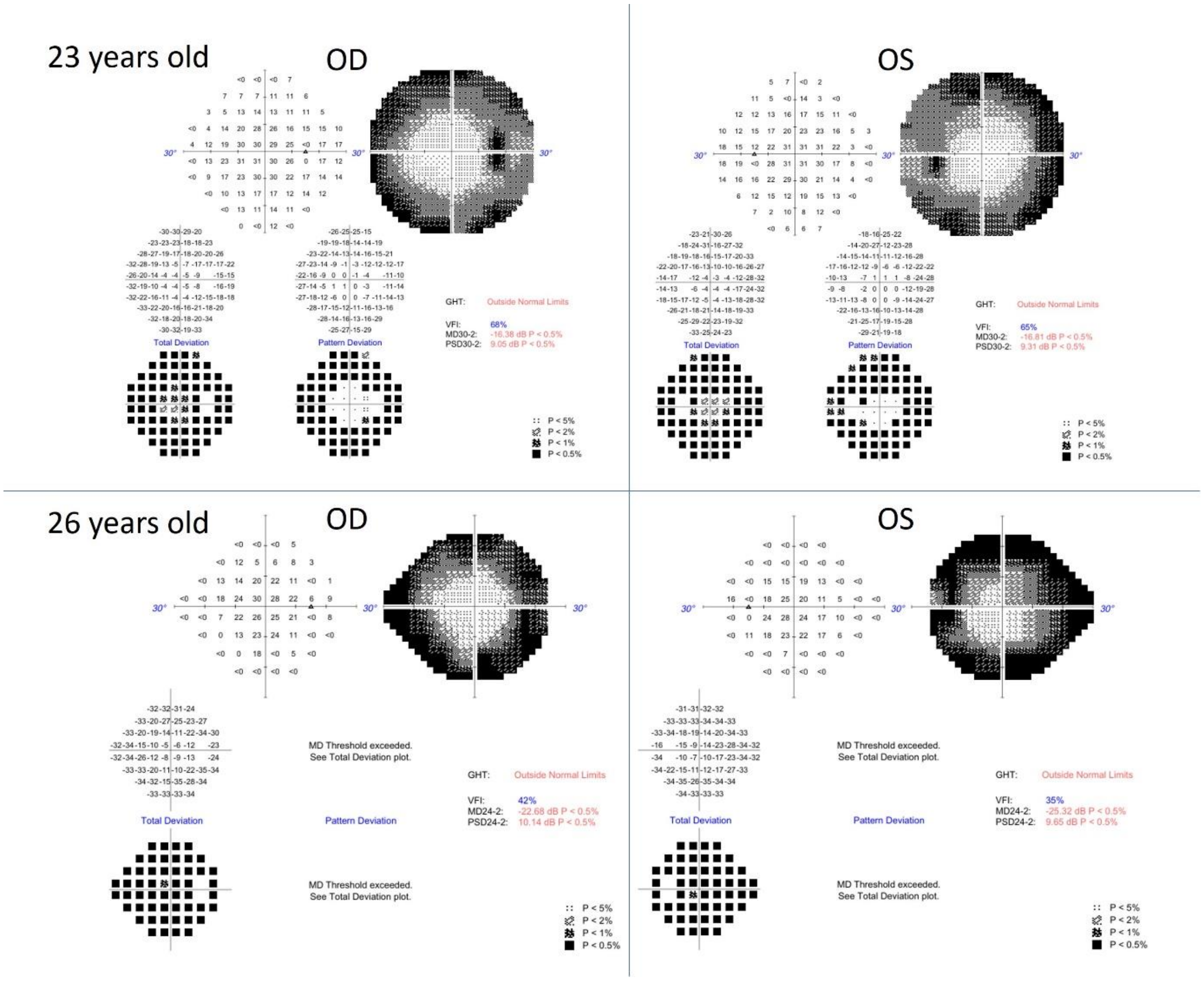

Fig. S1. Single Field Analysis of Visual Fields in Individual III-3 at ages 23 and 26. OD – right eye, OS – left eye. Classic visual field defects associated with RP: peripheral vision loss, evident as darkened areas on the perimetry map, indicating significant deficits in the retinal sensitivity typical for RP. Central vision is relatively preserved, highlighting the progressive nature of the disease.

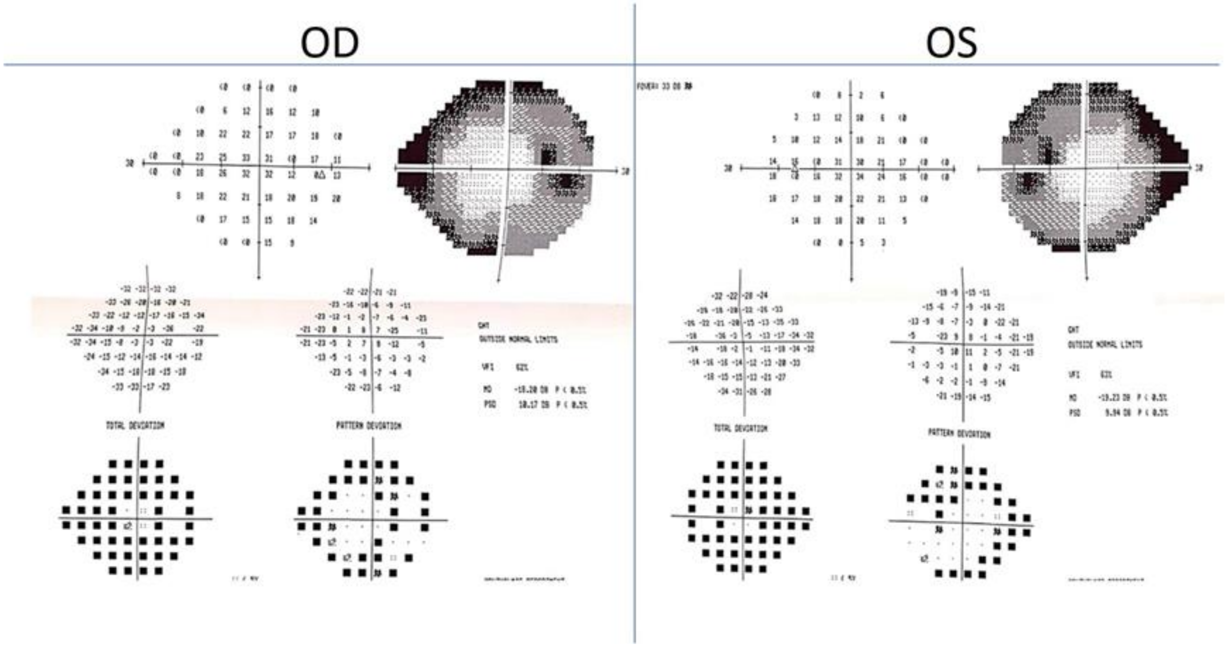

Fig. S2. Single Field Analysis of Visual Fields in Individual III-1 at age 16. OD – right eye, OS – left eye.

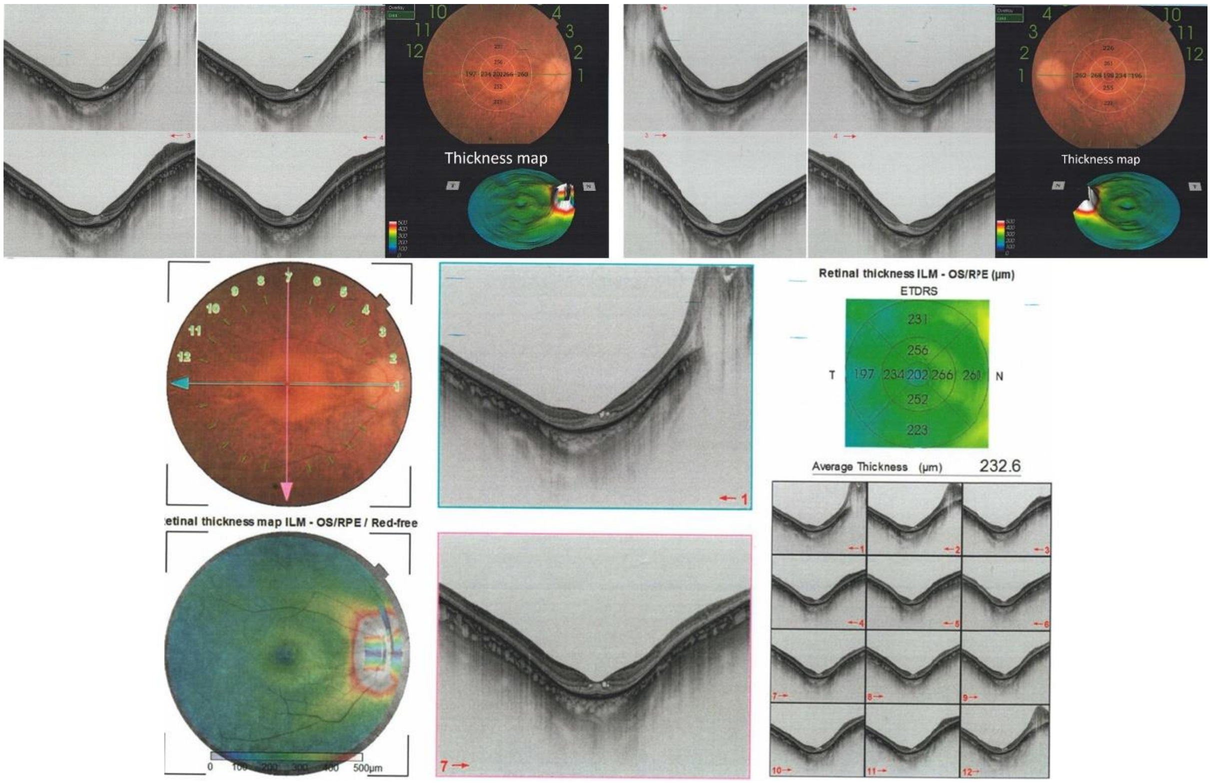

Fig. S3. Individual III-3 at age 26. Fundus photographs of both eyes, SD-OCT imaging, retinal thickness maps (upper part), and a colour photograph of the central retina with its corresponding SD-OCT scan of the right eye (lower part). Please note a pseudo-myopic appearance of the retina: although this individual has only mild myopia, advanced outer retinal thinning related to RP mimics morphology of high myopia, including a pseudo-conus.

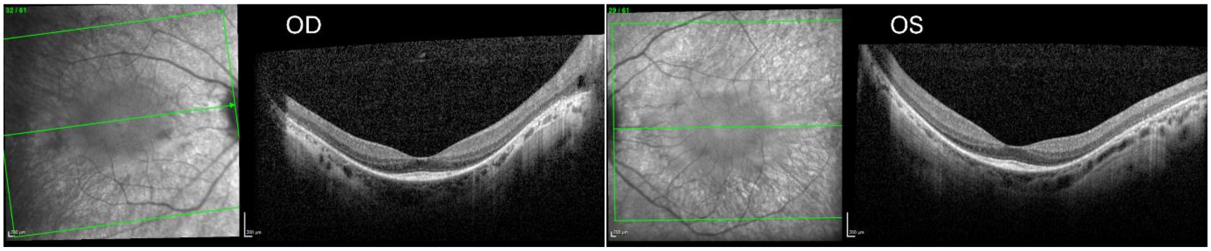

Fig. S4. Individual III-3 at age 28. OCT and fundus images of the right (OD) and left (OS) eyes. Severe retinal atrophy is evident, with almost complete loss of photoreceptors and retinal pigment epithelium layers, except in the central foveal depression.

ATGGTGAGCAAGGGCGAGGAGGATAACATGGCCATCATCAAGGAGTTCATGCGCTTCAAGGT
GCACATGGAGGGCTCCGTGAACGGCCACGAGTTCGAGATCGAGGGCGAGGGCGAGGGCCGC
CCCTACGAGGGCACCCAGACCGCCAAGCTGAAGGTGACCAAGGGTGGCCCCCTGCCCTTCGC
CTGGGACATCCTGTCCCCTCAGTTCATGTACGGCTCCAAGGCCTACGTGAAGCACCCCGCCGA
CATCCCCGACTACTTGAAGCTGTCCTTCCCCGAGGGCTTCAAGTGGGAGCGCGTGATGAACTT
CGAGGACGGCGGCGTGGTGACCGTGACCCAGGACTCCTCCCTGCAGGACGGCGAGTTCATCT
ACAAGGTGAAGCTGCGCGGCACCAACTTCCCCTCCGACGGCCCCGTAATGCAGAAGAAGAC
CATGGGCTGGGAGGCCTCCTCCGAGCGGATGTACCCCGAGGACGGCGCCCTGAAGGGCGAG
ATCAAGCAGAGGCTGAAGCTGAAGGACGGCGGCCACTACGACGCTGAGGTCAAGACCACCT
ACAAGGCCAAGAAGCCCGTGCAGCTGCCCGGCGCCTACAACGTCAACATCAAGTTGGACATC
ACCTCCCACAACGAGGACTACACCATCGTGGAACAGTACGAACGCGCCGAGGGCCGCCACTC
CACCGGCGGCATGGACGAGCTGTACAAGGTTAGCAAAGGTGAAGAACTGTTTACCGGAGTTG
TCCCCATTCTGGTCGAGCTTGATGGCGACGTGAATGGACACAAGTTTAGCGTAAGTGGAGAA
GGAGAAGGAGATGCCACGTATGGGAAACTGACCCTGAAGTTCATCTGCACAACTGGGAAATT
GCCTGTGCCTTGGCCGACTTTGGTGACAACCCTCACTTATGGCGTGCAGTGTTTCTCCAGATA
CCCCGATCACATGAAACAGCATGACTTCTTTAAGTCTGCCATGCCAGAAGGGTATGTACAGGA
ACGTACCATCTTCTTCAAAGATGACGGCAACTACAAGACGAGGGCTGAGGTGAAGTTTGAGG
GTGACACACTCGTCAATCGGATTGAGCTGAAAGGCATCGACTTTAAGGAGGATGGGAACATA
CTTGGTCACAAGCTGGAGTACgtaagtgccatgggattgggtggaaacctcgggcaagtacctttcccaaactcttctgggggtcca
tatttggagggaggggagagttctccgttgctctctctttccctcagccctgcctctgatcttcaatgcctttctcacttgt**G**gatgtccttcctgtcacgacag
AACTACAACTCCCATAACGTGTATATCATGGCTGACAAGCAGAAGAATGGCATAAAGGTGAAC
TTCAAAATCCGCCATAATATCGAGGATGGTTCAGTCCAACTCGCAGACCATTACCAGCAGAAC
ACTCCAATTGGCGATGGACCTGTGTTGCTTCCAGACAATCACTACCTGTCAACCCAATCTGCG
CTGAGCAAAGACCCCAATGAGAAACGAGATCACATGGTTCTGCTGGAGTTTGTGACAGCAGC
CGGGATTACCCTCGGCATGGACGAACTGTATAAGTAA

Fig. S5. Design of the Dual Fluorescence Splicing Reporter. The monomeric derivative of DsRed fluorescent protein is shown in red, while the split eGFP (containing 5' and 3' portions) is shown in green. The split eGFP segments are interrupted by the entire 172 bp *PRPF3* wild-type intron 11 (represented in lowercase letters). A capital G in bold within the intron indicates the position of the studied G>A variant.

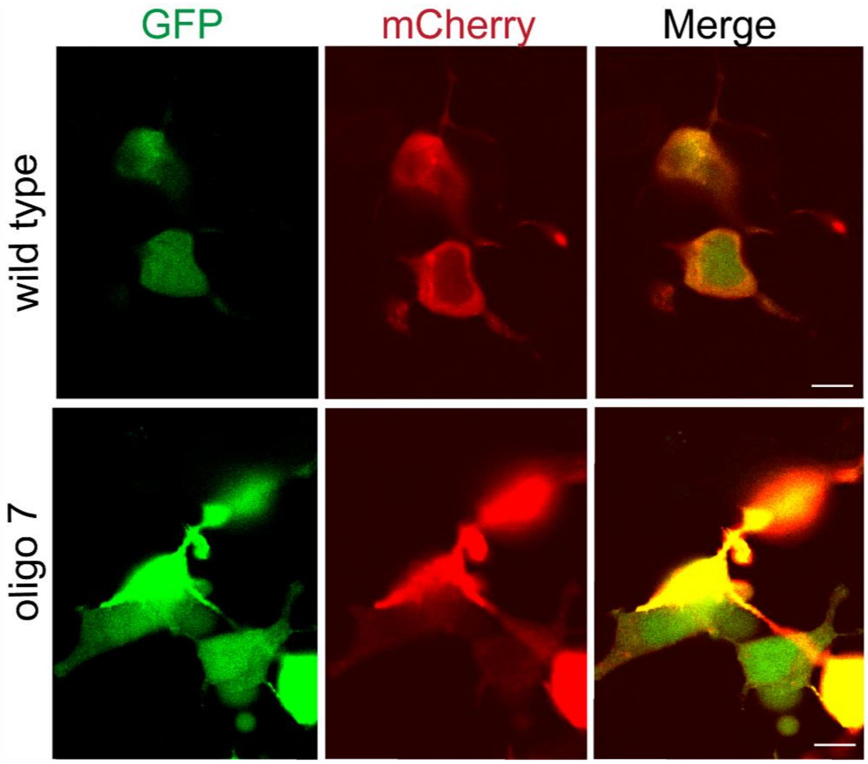

Fig. S6. Confocal microscopy of HEK293T cells transfected with the WT and mutated constructs, alongside AON-7 (750 ng). Merging red and green fluorescence images resulted in yellow signals, indicating co-expression of dual fluorescence in the wild-type samples and splicing restoration of AON-7. Scale bar, 10 µm.
